# Autoimmune encephalitis with psychiatric features in adults: historical evolution and prospective challenge

**DOI:** 10.1007/s00702-020-02258-z

**Published:** 2020-10-07

**Authors:** Niels Hansen, Charles Timäus

**Affiliations:** Department of Psychiatry and Psychotherapy, University of Goettingen, Von-Siebold-Str. 5, 37075 Goettingen, Germany

**Keywords:** Autoimmune encephalitis, Psychiatry, Autoantibodies, Intracellular antibodies, Membrane surface antibodies

## Abstract

Our review aims to delineate the psychiatric spectrum of autoantibody-associated autoimmune encephalitis over time through its discoveries of antibodies. We searched in PubMed for appropriate articles depicting the first appearance and spectrum of psychiatric symptomatology in autoantibody-positive encephalitis for this narrative review. Memory impairment was first associated with autoantibodies against intracellular antigens such as anti-HuD antibodies in 1993. 8 years later, autoantibodies against cell membrane surface antigens such as voltage-gated potassium channels were described in conjunction with memory dysfunction. The spectrum of psychiatric syndromes was amplified between 1990 and 2020 to include disorientation, behavior, cognitive dysfunction, obsessive compulsive behavior and suicidality in encephalitis patients occurring together mainly with antibodies against surface antigens, less so against intracellular antigens. In general, we found no specific psychiatric symptoms underlying specific autoantibody-associated encephalitis. As fundamental data on this issue have not been systemically assessed to date, we cannot know whether our specific findings would remain from systematic studies, i.e., on the association between cerebrospinal fluid N-methyl-D-aspartate receptor antibodies in catatonia. The psychiatric symptomatology overlaps between psychiatric domains and occurs frequently in antibody-positive encephalitis. No specific psychiatric symptoms imply an underlying, specifically autoantibody-associated encephalitis. The psychiatric phenotypology associated with antibody-positive encephalitis has evolved tremendously recently, and this new evidence reveals its relevance for future diagnostic and treatment aspects of autoimmune encephalitis patients.

## Introduction

Autoimmune encephalitis is a disorder that can dynamically alter its phenotypical appearance over time. It is often characterized by an initial psychiatric manifestation, or reveals predominant or isolated psychiatric features (Kayser et al. 2013; Herken and Prüss 2017). The aim of this review is to depict the historic evolution of the published psychiatric phenomenology of autoimmune encephalitis.

## Methods

We looked through the PubMed database for appropriate articles comprising the terms “autoimmune encephalitis AND psychiatry”, “AK5 (adenylate kinase 5)/Amiphiphysin/AMPAR (ɑ-amino-3-hydroxy-5-methyl-4-isoxazolepropionic acid receptor)/ BRSK2 (BR serine/threonine kinase 2)/CASPR2 (contactin-associated protein 2/CRMP3/4 (collapsing reponse mediator protein 3/4)/CV2/CRMP5 (cronveinten 2/collapsing response mediator protein 5), debrin/ DPPX (dipeptidyl aminopeptidase-like protein 6), GABAAR (gammaaminobutyric acid protein A receptor)/GABABR (gammaaminobutyric acid protein B receptor)/GAD65 (glutamic acid decarboxylase 65)/GlycinR/HuD/KLP11 (Kelch like 11 protein)/LGI1 (Leucine rich glioma inactivated protein 1)/Ma/Ta/mGluR5 (metabotropic glutamate receptor 5)/Neurexin3alpha/NMDAR (N-methyl-D-aspartate receptor)/Ri, Ro, SOX1/Synapsin/VGKC (voltage gated potassium channel)/Zic4, AND psychiatry”. We elucidate the autoantibodies’ discoveries via their first psychiatric presentation. Our classification of psychiatric features relies on the analysis strategy of Al Diwani et al. (2019), categorizing psychiatric features into eight main domains entailing dysfunctional behavior, catatonia, eating or mood abnormalities, obsessive–compulsive behavior, psychosis, sleep dysfunction and suicidality.

## Results

### First description of presumed paraneoplastic encephalitis with psychiatric features

**Fig. 1:**
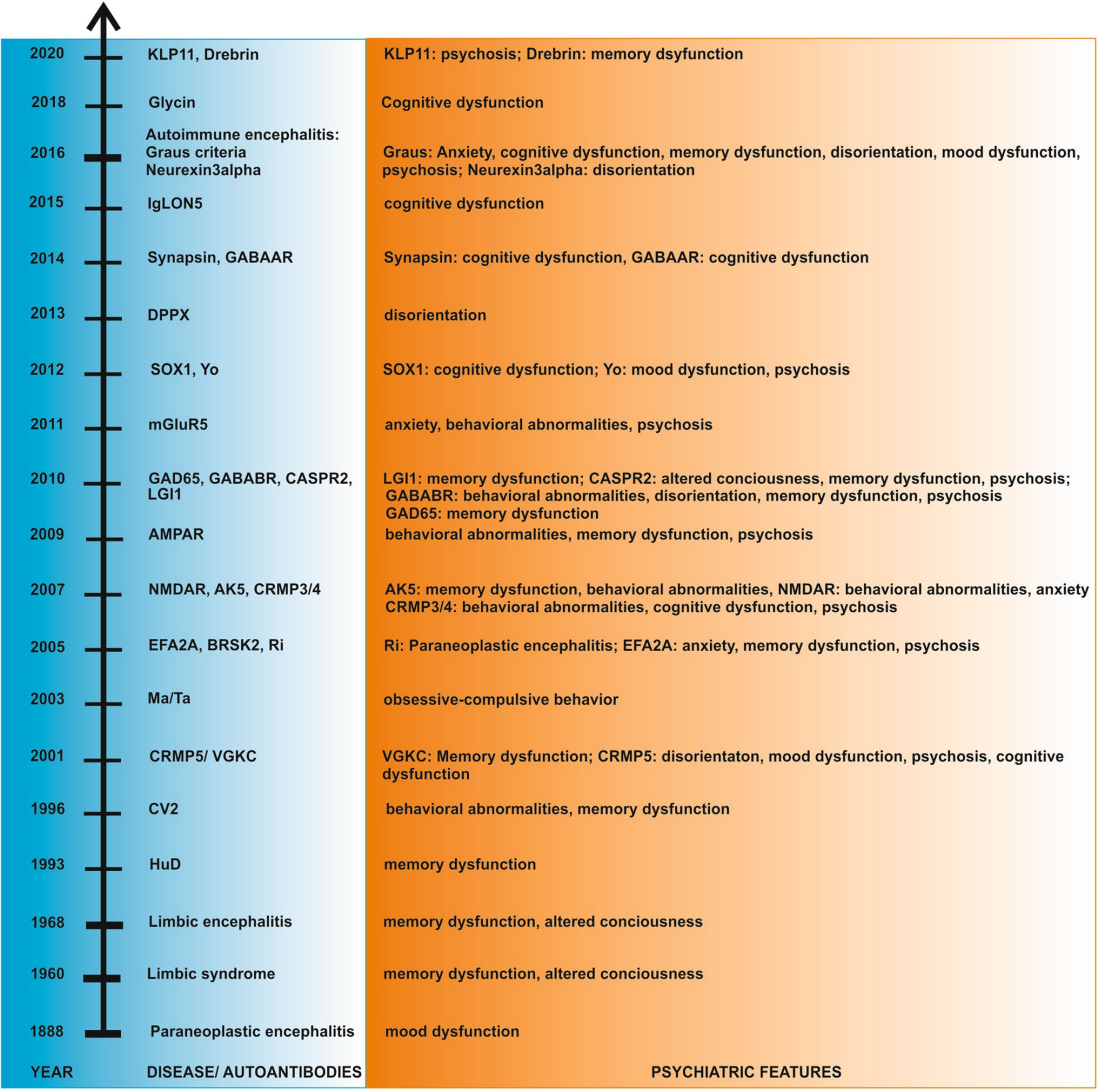
Timeline of autoimmune encephalitis with psychiatric features. *AE-GC* autoimmune encephalitis-Graus criteria, *AK5* adenylate kinase 5, *AMPAR* ɑ-amino-3-hydroxy-5-methyl-4-isoxazolepropionic acid receptor, *B* behavior, *BRSK2* BR serine/threonine kinase 2, *CASPR2* contactin-associated protein 2, *CRMP3/4* collapsing reponse mediator protein 3/ 4, *CRMP5* collapsing response mediator protein 5, *CV2* cronveinten 2, *DPPX* dipeptidyl aminopeptidase-like protein 6, *GABAAR* gammaaminobutyric acid protein A receptor, *GABABR* gammaaminobutyric acid protein B receptor, *GAD65* glutamic acid decarboxylase 65, *KLP11* Kelch like 11 protein, *LGI1* Leucine-rich glioma-inactivated protein 1, *mGluR5* metabotropic glutamate receptor 5, *NMDAR* *N*-methyl-D-aspartate receptor, *VGKC* voltage-gated potassium channel

Neuroimmunological researchers suggest (Schulz and Prüss 2015) that paraneoplastic autoimmune encephalitis was first described over 130 years ago by the neurologist Oppenheim 1888 (Fig. 1). Oppenheim described a 54-year-old woman with neuropsychiatric symptomatology along with a prominent mood dysfunction in addition to other brain dysfunctions such as aphasia and agnosia. Her autopsy revealed gastric cancer, but no abnormalities were observed in brain tissue. Thus it was postulated that the tumor could be the cause of focal neurological symptoms. The presumption of carcinoma-induced neurological symptoms implies a possible autoimmune process induced by tumor immunity.

### First description of limbic encephalitis with psychiatric features

About 80 years later, Brierley described three patients with limbic encephalitis comprising psychiatric features such as memory impairment, depressive syndrome, behavioral abnormalities and anxiety in addition to seizures and disturbed consciousness (Brierley et al. 1960) (Fig. 1). The notion limbic encephalitis was named nearly a decade later by Corsellis (Corsellis et al. 1968) (Fig. 1). Limbic encephalitis corresponds to an autoimmune encephalitis in the limbic system space. “Limbic syndrome” entails several specific clinical features such as memory impairment, seizures, and psychiatric abnormalities.

### Consensus criteria for autoimmune encephalitis including psychiatric features

Nearly 50 years later, Graus et al. (2016) (Fig. 1) developed consensus criteria to define autoimmune encephalitis. Its definition is based on four criteria—all of which are necessary for this diagnosis. The four criteria are: (1) subacute onset of symptoms characterized by “working memory deficits, seizures, or psychiatric symptoms”. The symptoms must reveal the tendency to accelerate over a timeline lasting under 3 months. Furthermore, either “working memory deficits, seizures, or psychiatric symptoms” together with limbic-system involvement must be present. In addition, (2) magnetic resonance imaging should depict brain abnormalities in both hemispheres within the medial temporal lobes on magnetic resonance imaging (MRI). Furthermore, (3) CSF should reveal pleocytosis or the EEG should show abnormal temporal activity as epileptic potentials or slowing. (4) Several alternative possible causes should be excluded.

In contrast, the clinician should assume a possible autoimmune encephalitis with psychiatric features according to Graus et al. (2016) if three criteria are met. The first criterion encompasses a subacute onset entailing changes in mental status and psychiatric symptoms, in addition to working memory deficits. The second criterion comprises one of the following: novel central focal neurological deficits, novel seizures, pleocytosis in CSF, or unilateral MRI temporal abnormalities. The third criterion is the absolute exclusion of any other possible causes. Two main subgroups of autoimmune encephalitis are distinguished according to their associated autoantibodies: those with autoantibodies (1) directed against membrane surface antigens and those (2) that target intracellular antigens. In our review, we employ the superordinate term autoimmune encephalitis, although most reports refer to a limbic encephalitis as a subform of autoimmune encephalitis–but the superordinate concept included also NMDAR encephalitis often not restricted to the limbic system.

### Differential diagnosis of autoimmune encephalitis with psychiatric features

The differential diagnosis of autoimmune encephalitis is often demanding, and other diseases must be carefully ruled out in every patient. Several other disease entities need to be considered, such as the neuropsychiatric phenotype of lupus erythematosus often associated with double-stranded deoxyribonucleic acid antibodies, anti-antiphospholipid antibodies, anti-ß2-glycoprotein-I, lupus anti-coagulant or anti-ribonucleoprotein antibodies (Nikolopoulos et al. 2020). Furthermore, Hashimoto encephalopathy in conjunction with psychiatric symptoms and thyroid autoimmunity must be excluded by thoroughly investigating the existence of antibodies against thyroid peroxidase (TPO) and thyroglobulin (TG) (Barbero et al. 2019; Barbuti et al. 2017). In children, it is important to screen for pediatric autoimmune neuropsychiatric disorder, which is associated with streptococcal infections (PANDAS) entailing a tic disorder together with preceding streptococcal infections revealing antibodies against streptococcal proteins, human brain enolase or neural tissue (Nicollini et al. 2015; Shimasaki et al. 2020). Another neuropsychiatric syndrome occurring in children and young adults is Rasmussen encephalitis: a chronic neuroinflammation of one hemisphere along with drug-resistant seizures and severe cognitive dysfunction (Varadakar et al. 2014). These disease entities need to be carefully excluded during the differential diagnosis before specifying the autoimmune encephalitis diagnosis.

### Autoimmune encephalitis associated with antibodies against intracellular antigens

Historically speaking, autoimmune encephalitis with antibodies against intracellular antigens was discovered before autoimmune encephalitis with antibodies against membrane surface antibodies. The first report of HuD-positive encephalitis described a memory impairment as a relevant neuropsychiatric appearance (Tsukamato et al. 1993) (see Fig. 1 for autoimmune encephalitis associated with specific autoantibodies and its first psychiatric presentation; see Table 1 for psychiatric syndromes associated with autoantibodies). HuD is an intracellular RNA-packed protein that is important for nervous system upgrowth and synaptic plasticity in the brain (Perrone-Bizzozero and Bird 2013). It is thus not surprising that autoantibodies against HuD affect synaptic transmission, thereby inducing memory impairment; however, the exact mechanism is unclear. Later, Graus confirmed in 2001 (Graus et al. 2001) that memory impairment is a cardinal feature of HuD-positive encephalitis. Two years later, other clinical psychiatric features were described such as topographical disorientation in a 70-year-old woman (Hirayama et al. 2003) (Fig. 1). In 1996, another paraneoplastic antibody against the intracellular antigen CV2 was identified in paraneoplastic neurological syndromes including encephalitis partly characterized by psychiatric features in 45 patients. Three of those patients had a limbic encephalitis presenting with catatonic behavior such as mutism and amnesia (Honnorat et al. 1996). The 66 kDa peptide CV2/CRMP5 is often expressed in the thymus and within a thymoma (Camdessanché et al. 2006), indicating their impact on immunity and in regulating tumor immunity. In 2001–2002, cognitive impairment, disorientation, depressive and psychotic syndrome were reported as the clinically symptomatological complex of CRMP5-positive encephalitis (Yu et al. 2001; Vernino et al. 2002) (Fig. 1). Obsessive–compulsive behavior can also be a manifestation of this disease (Muehlschlaegel et al. 2005). Obsessive–compulsive behavior in association with Ma/Ta-positive encephalitis has also been identified (Scheid et al. 2003) (Fig. 1). A year later, Dalmau et al. (2004) reported a more variable clinical picture ranging from delirium to depressive syndrome in a larger cohort of 38 patients with Ma2-positive encephalitis. Four years later, personality changes, amnesia or behavioral symptoms such as lethargy were observed in 22 patients with Ma/Ta paraneoplastic limbic encephalitis (Hoffmann et al. 2008). A man was reported in 2005 presenting with encephalitis due to serum Ri antibodies based on a lung carcinoma associated with personality changes and neuropsychological deficits (Harloff et al. 2005) (Fig. 1). Another novel autoantigen was discovered in 2005 (Fig. 1) by Sabater’s group (Sabater et al. 2005), who diagnosed serum BR serine/threonine kinase 2 (BRK2) antibodies in a patient with a “limbic syndrome” in conjunction with lung cancer crucial for neuronal transmission and development. As there have been no further reports on this antibody, it must occur very seldom in autoimmune encephalitis patients. Two years later, serum and CSF adenylate kinase 5 antibodies were discovered in two men with autoimmune encephalitis associated with memory deficits, personality changes, and behavioral symptoms such as aggression and agitation (Tüzün et al. 2007) (Fig. 1). Their role in cellular homeostasis, and the particular expression of AK5 in the brain constitutes neuronal dysfunction within brain homeostasis if CSF AK5 antibodies are detected. Later reports of other psychiatric features were published, such as behavioral symptoms like disinhibition and memory loss (Ng et al. 2015) including anterograde amnesia (Do et al. 2017; Bien et al. 2019), mood disturbances including a depressive syndrome (Do et al. 2017; Bien et al. 2019), and anxiety (Do et al. 2017). The specific pathophysiological relevance of cross-reactive antibodies is unclear. In patients with encephalitis and HuD antibodies as well as serum and CSF SOX1 antibodies (Fig. 1) were reported in a 59-year-old female suffering from cognitive impairment (Stich et al. 2012), recalling that the contribution of SOX1 antibodies to cognitive impairment remains unknown. A schizoaffective syndrome was reported in 2012 as being associated with low-titer serum and CSF paraneoplastic Yo antibodies directed against an intracellular 62 kDa Purkinje cell protein (Fig. 1). Neuroimaging revealed no typical signs of encephalitis, but frontotemporal atrophy in MRI and cerebellar hypometabolism in FDG-PET were diagnosed (Endres et al. 2015). We suspect that a chronic neuroinflammation might have led to neurodegeneration culminating in frontotemporal atrophy. Novel serum Drebrin autoantibodies have been detected in patients with suspected limbic encephalitis, often accompanied by memory impairment (Pitsch et al. 2020) (Fig. 1). Drebrin, the postsynaptic protein located intracellularly, plays an important role in synaptic function and hippocampal excitability (Pitsch et al. 2020); therefore, the memory impairment we observe in these patients might result from disturbed synaptic neurotransmission and altered neuronal excitability in the hippocampus.

**Table 1:** Autoimmune encephalitis with psychiatric syndromes and symptoms associated with autoantibodies in adults

Psychiatric symptoms/syndromes	ABS associated	References
Serum autoantibodies		
Disorientation	AMPAR, CASPR2, CRMP5, GABABR, DPPX, HuD, NMDAR, neurexin3alpha, Ma2	Boronat et al. 2013; Dalmau et al. 2004; Si et al. 2019; Gresa-Arribas et al. 2016; Hirayama et al. 2003; Joubert et al. 2016; Tsukamato et al. 1993; Vernino et al. 2002; Vincent and Irani 2010; Yu et al. 2001
Cognitive and memory dysfunction	AK5, AMPAR, CASPR2, CRMP3/4, CV2, DPPX, drebrin, GABAA/BR GAD65, glycineR, HuD, IgLON5, KLP11, LGI1, Ma/Ta, mGluR5, NMDAR, Ri, SOX1, VGKC	Bien et al. 2019; Dalmau et al. 2008; Do et al. 2017; Dogan Onugoren et al. 2015; Finke et al. 2012; Hansen et al. 2020a; Hara et al. 2017; Harloff et al., et al. 2005; Hoffmann et al. 2008; Honnorat et al. 1996; Knudsen et al. 2007; Loane et al. 2019; Malter et al. 2010; Maudes et al. 2020; Pitsch et al. 2020; Spatola et al. 2017, 2018; Stich et al. 2012; van Sonderen et al. 2016; Swayne et al. 2018; Tsukamoto et al.^1993^; Yu et al. 2001
Behavior	AK5, AMPAR, CRMP3/4, DPPX, GABAA/BR, HuD, mGluR5, neurexin3alpha, Ma/Ta, NMDAR, synapsin	Boronat et al. 2013; Do et al. 2017; Gresa-Arribas et al. 2016; Hoffmann et al. 2008; Honnorat et al. 1996; Hoffmann et al. 2008; Lai et al. 2009; Lancaster et al. 2019; Saether et al. 2019; Samra et al. 2020; Spatola et al. 2017, 2018
Psychosis	AMPAR, CASPR2, CRMP5, EFA2A, glycin R, KLP11, LGI1, mGluR5, NMDAR, synapsin, Yo, VGKC	Dalmau et al. 2008; Endres et al. 2015; Kruse et al. 2015; Piquet et al. 2019; Saether et al. 2019; Vitilani et al. 2005; Warren et al. 2019; Wang et al. 2018; Yu et al. 2001
Mood dysfunction	AK5, AMPAR, CRMP5, EFA2A; GAD65, NMDAR, Ma2, synapsin, VGKC	Bien et al. 2019; Dalmau et al. 2004; Hansen et al. 2016; Höftberger et al. 2015; Kruse et al. 2015; Saether et al. 2019; Vernino et al. 2002; Vitilani et al. 2005; Yu et al. 2001
Anxiety	AK5, EFA2A, GAD65, mGluR5, NMDAR	Do et al. 2017; Spatola et al. 2018; Sansing et al. 2007; Vitilani et al. 2005
Catatonia	NMDAR, GABAAR	Finke et al. 2012, Samra et al. 2020
Sleep dysfunction	CASPR2, DPPX, EFA2A, IgLON5, mGluR5, NMDAR	Boronat et al. 2013; Finke et al. 2012; Spatola et al. 2018; Van Sonderen et al. 2016, Vitilani et al. 2005
Obsessive–compulsive behavior	Ma/Ta	Scheid et al. 2003
Suicidality	VGKC, mGluR5	Kruse et al. 2015; Lancaster et al. 2011
CSF autoantibodies		
Disorientation	AMPAR, GABABR, neurexin 3alpha, Ma2, synapsin	Dalmau et al. 2004; Lai et al. 2009; Si et al. 2019; Piepgras et al. 2015
Cognitive and memory dysfunction	AK5, AMPAR, CASPR2, GABAAR, GABABR, GAD65; LGI1, mGluR5, NMDAR	Dalmau et al. 2008; Do et al, 2017; Finke et al. 2012; Guevara et al. 2018; Hansen et al. 2016; Lai et al. 2009, 2010; Spatola et al. 2017, 2018; Van Sonderen et al. 2016
Behavior	AK5, AMPAR, GABAAR, mGluR5, neurexin 3 alpha, NMDAR	Do et al. 2017; Gresa-Aribas et al. 2016; Höftberger et al. 2013; Khadem et al. 2009; Lai et al. 2009; Spatola et al. 2018
Psychosis	AMPAR, CASPR2, EFA2A, glycine R, KLP11, mGluR5, NMDAR, VGKC	Dalmau et al. 2008; Höftberger et al. 2015; Joubert et al. 2016; Maudes et al. 2020; Spatola et al. 2018; Vincent et al. 2004; Vitaliani et al. 2005
Mood dysfunction	AK5, AMPAR, EFA2A, GAD65, LGI1, Ma2, NMDAR	Bien et al. 2019; Kopczak et al. 2017; Dalmau et al. 2004; Hansen et al. 2016; Höfterger et al. 2015; Kayser et al. 2013; Vitaliani et al. 2005
Anxiety	AK5, EFA2A, mGluR5, NMDAR	Do et al. 2017; Maat et al. 2013; Spatola et al. 2018; Tu et al. 2018; Vitaliani et al. 2005
Catatonia	NMDAR	Khadem et al. 2009
Sleep dysfunction	AMPAR, CASPR2, IgLON5, mGluR5, NMDAR	Finke et al. 2012; Lai et al. 2009; Spatola et al. 2018; Van Sonderen et al. 2016

*ABS* autoantibodies, *AK5* adenylate kinase 5, *AMPAR* ɑ-amino-3-hydroxy-5-methyl-4-isoxazolepropionic acid receptor, *BRSK2* BR serine/threonine kinase 2, *CASPR2* contactin-associated protein 2, *CRMP3/4* collapsing reponse mediator protein 3/4, *CRMP5* collapsing response mediator protein 5, *CV2* cronveinten 2, *DPPX* dipeptidyl aminopeptidase-like protein 6, *GABAAR* gammaaminobutyric acid protein A receptor, *GABABR* gammaaminobutyric acid protein B receptor, *GAD65* glutamic acid decarboxylase 65, *KLP11* Kelch-like 11 protein, *LGI1* leucine-rich glioma-inactivated protein 1, *mGluR5* metabotropic glutamate receptor 5, *NMDAR* *N*-methyl-D-aspartate receptor, *VGKC* voltage-gated potassium channel

### Autoimmune encephalitis together with autoantibodies against membrane surface antigens

Patients with autoimmune encephalitis associated with antibodies against membrane surface antigens are more often affected by psychiatric symptoms than are those with autoimmune encephalitis associated with autoantibodies against intracellular antigens (Fig. 1; Table 1).

#### VGKC antibody-positive encephalitis

Limbic encephalitis associated with limbic mental symptoms such as memory disturbances have been already reported by Buckley (2001) in two patients presenting with positive serum VGKC antibodies (Fig. 1). Just 3 years later, the psychiatric spectrum was expanded to include behavioral abnormalities such as apathy, mood symptoms like irritability, and psychotic symptoms such as visual hallucinations reportedly associated with VGKC-positive encephalitis (Thieben et al. 2004; Vincent et al. 2004). Vincent et al. (2004) described psychiatric symptoms in VGKC autoimmunity not associated with tumors, highlighting the concept of non-paraneoplastic autoimmunity. The psychiatric spectrum became amplified 10 years later as personality changes, depressive syndrome and anxiety in 67 patients with VGKC serum antibodies. Depression and anxiety were observed in 62% of these patients (Somers et al. 2011). Another investigation reported mood dysfunction and anxiety in 17% of 316 patients with VGKC antibodies (Klein et al. 2013). Cognitive dysfunction was more deeply investigated by Butler et al. (2014), who found that some patients with VGKC encephalitis exhibited relevant memory impairment, decelerated processing, and executive dysfunction. Furthermore, some patients revealed persistent anterograde amnesia even when other symptoms were immunotherapy responsive. In a smaller cohort of six patients with VGKC antibody-positive probable autoimmune encephalitis, other distinct psychiatric features such as suicidal thoughts, mania or psychosis was observed (Kruse et al. 2015). In their recent review, Prüss and Lennox (2016) analyzed 13 patients with neuropsychiatric symptoms who revealed additional psychiatric symptoms such as mood and cognitive dysfunction, as well as sleep disturbances.

#### NMDAR antibody-positive encephalitis

The most prevalent (and often diagnosed as “psychiatric”) encephalitis is associated with NMDAR antibodies. In NMDAR encephalitis, the psychiatric symptoms even precede the appearance of neurologic symptoms (Dalmau et al. 2019). A typical constellation is an abrupt onset of psychiatric symptoms such as delusions, amnesia or agitation with no prior psychiatric history (Marinova et al. 2019). In Sansing et al. (2007) described a woman who exhibited a variety of psychiatric symptoms such as mainly behavioral transformations such as aggression and agitation as well as anxiety and even homicidal ideation (Fig. 1). A large cohort of 100 patients was described a year later by Dalmau (2008). All of their patients presented with psychiatric symptoms including memory disturbances (they unfortunately failed to characterize the psychiatric symptoms further). Other single case reports augmented the psychiatric spectrum potentially associated with NMDAR encephalitis. A woman with NMDAR encephalitis displayed catatonia in 2009 (Khadem 2009), a symptom that has since then often been considered as pathognomonic for NMDAR encephalitis (Espinola-Nadurille et al. 2019). In NMDAR encephalitis, patients often present a catatonic syndrome including delirium, psychomotor agitation, and hallucinations; immunotherapy has delivered very promising results (Espinola-Nadurille et al. 2019). The first report of an acute psychosis was published in 2010 (Fawcett et al. 2010) and later confirmed by others (Tidswell et al. 2013). In their large observation group of 571 patients, Kayser et al. (2013) explored the spectrum of isolated psychiatric symptoms found in 4% of NMDAR-positive encephalitis patients. This spectrum comprised behavioral symptoms such as aggression, disinhibition and suicidality, psychotic syndrome comprising delusions, mood changes including mania and depression, and catatonic symptoms such as echolalia (referring to classification Al-Diwani et al. (2019)) and catatonia. The most frequent symptoms were mood dysfunction in 70%, delusions in 74%, aggression in 57% and auditory or visual hallucinations in 43% (Kayser et al. 2013). 83% of these patients recovered following immunotherapy or tumor removal, as had been diagnosed in 43% (Kayser et al. 2013). Schizophreniform psychosis is a frequent phenomenon and has been supported by a recent study of a 60-patient cohort of patients suffering first-episode psychosis and additional neurological features such as seizures or autonomic symptoms [27% of these patients had been diagnosed with NMDAR encephalitis] (Tang et al. 2019). The complex psychiatric presentation of NMDAR encephalitis was enriched in subsequent years to incorporate bipolar mood dysfunction and mania (Choe et al. 2013; Fousse et al. 2013), anxiety (Maat et al. 2013) and behavioral abnormalities including agitation (Maat et al. 2013). Psychotic and depressive syndrome often coexist, as reported by a large-scale study of Al-Diwani et al. (2019) including 505 patients reported with psychiatric symptomatology in NMDAR encephalitis.

#### AMPAR-positive encephalitis

The autoantibody against the AMPAR receptor was reported in ten patients with limbic encephalitis in 2009 (Fig. 1). Antibodies against AMPAR might impair learning and memory, as a recent in vitro and in vivo study in mice demonstrated (Haselman et al. 2008). Their patients revealed a phenotype consisting of behavioral abnormalities including agitation and lethargy, short-term memory dysfunction, confabulation, disorientation, hallucinations and insomnia (Lai et al. 2009). Applying these antibodies to neuronal cultures reduced the size of AMPAR aggregations at the synapse (Lai et al. 2009), suggesting synaptic dysfunction as a probable pathophysiological basis of various psychiatric symptoms such as memory dysfunction induced by AMPAR antibodies (O´Reilly et al. 2019). Another case report indicated that rapid cycling bipolar symptoms could also suggest AMPAR encephalitis (Quaranta et al. 2015).

#### LGI1-positive encephalitis

Another antibody is directed against the LGI1 antigen, which is part of the VGKC complex interacting with presynaptic ADAM metallopeptidase domain 23 (ADAM23) and postsynaptic ADAM metallopeptidase domain 22 (ADAM22). LGI1 antibodies might induce neuronal apoptosis, as recently demonstrated in hippocampal neurons (Aysit-Altuncu et al. 2018), potentially leading to irreversible neuronal dysfunction and thus diverse symptoms such as episodic memory dysfunction. Memory disturbances are a frequent symptom, reported in 57 patients with serum and CSF VGKC antibodies (Lai et al. 2010) (Fig. 1). Memory impairment in LGI1 patients was related to stronger functional connectivity with the insula brain region, salience network, and default mode network, indicating network-based impairments due to LGI1 encephalitis (Heine et al. 2018). Over the last 5 years, more psychiatric symptoms such as depressive syndrome (Murata et al. 2015), mania (Tu et al. 2018) and psychosis (Wang et al. 2018) have been described as manifesting as the main clinical presentations. These psychiatric manifestations may be attributable to impaired connectivity in those brain regions involved in motion and cognition (Qiao et al. 2020).

#### CASPR2-positive encephalitis

In the same year that CASPR2 antibodies were identified as also being part of the VGKC complex in patients with LGI1 encephalitis, patients with CASPR2-positive encephalitis were clinically characterized by confusion, amnesia, and hallucinations (Vincent and Irani 2010) (Fig. 1). In patients with CASPR2 CSF-positive encephalitis, memory dysfunction was a primordial feature of the phenotypology (Joubert et al. 2016). CASPR2 encephalitis’ psychotic phenomenology was recently reported in conjunction with postpartum psychosis (Warren et al. 2019).

#### GABAAR- and GABABR-positive encephalitis

In addition to CASPR2 LGI1 encephalitis, further autoantibodies termed GABABR antibodies were reported in 2010 to be associated with memory dysfunction, confusion, behavioral abnormalities, and psychosis (Lancaster et al. 2010) (Fig. 1). Although GABABR-positive encephalitis’ pathophysiology is still not well understood, a study revealed that CD8 + T cells are major players in GABABR-positive encephalitis (Golombeck et al. 2016). GABAAR-antibody encephalitis was discovered 4 years later in patients presenting with cognitive impairment (Petit Pedrol et al. 2014) (Fig. 1). The phenotype was characterized by episodic memory dysfunction, confusion, agitation, and catatonic features such as echolalia and mutism (Si et al. 2019; Samra et al. 2020). (Al Diwani’s classification of psychiatric features (Al Diwani et al. 2019)).

#### GAD65-positive encephalitis

2010 temporal lobe epilepsy due to limbic encephalitis associated with serum and CSF GAD65 antibodies was first described by Malter in a small cohort of patients (Malter et al. 2010) (Fig. 1). Stiff person autoimmune serum autoantibodies suffice, but for others, like autoimmune encephalitis, intrathecal GAD65 is required to confirm autoimmunity (Graus et al. 2020). GAD65-positive encephalitis was diagnosed in a woman with depressive syndrome, cognitive impairment, and autoimmune polyendocrinopathy–candidiasis–ectodermal dystrophy (APECED) syndrome diagnosed after detecting CSF antibodies (Kopczak et al. 2017) and confirming their relevance to autoimmunity. Mood dysfunction and cognitive impairment are frequent symptoms in GAD65-positive encephalitis (Hansen et al. 2016; Hansen et al. 2018). Furthermore verbal and figural memory deficits are often diagnosed in conjunction with GAD65 encephalitis over long time intervals (Hansen et al. 2018). The morphological basis for severe disturbances in emotional regulation is probably the amygdala’s altered volume and signal intensity that GAD65-positive patients with limbic encephalitis have revealed (Wagner et al. 2015).

#### Autoimmune encephalitis subtypes associated with autoantibodies against membrane surface antigens

In 2005, the serum and CSF autoantibody against a protein termed EFA6A (that interacts with potassium channels) was reported to be associated with a paraneoplastic encephalitis associated with a depressive syndrome, incoherent thoughts, insomnia, panic, personality changes and psychotic symptoms such as delusional thinking and auditory hallucinations (Vitaliani et al. 2005) (Fig. 1). An encephalitis subtype associated with serum CRMP3/4 antibodies was first reported in 2007 (Kudsen et al. 2007) (Fig. 1). Its symptomatology ranges from cognitive decline to agitation and hallucinations. This antigen protein is involved in nerve neural growth. CRMP3/4 antibodies were stained in synaptic pyramidal cells and granule cells in the hippocampus. Due to the key role the hippocampus plays in memory and cognition, these CRMP3/4 antibodies—identified on hippocampal pyramidal and granule cells—might explain the aforementioned cognitive dysfunction. Four years later, two patients with limbic encephalitis presenting with serum mGluR5-receptor antibodies were determined suffering from fear, agitation, auditory and visual hallucinations, and suicidal thoughts (Lancaster et al. 2011) (Fig. 1). Later, the spectrum of psychiatric symptoms was expanded to include inattention and anterograde memory impairments (Guevara et al. 2018). This variable psychiatric spectrum including memory dysfunction and anxiety might result from mGLUR5 involvement in synaptic transmission and neuromodulatory control over neuronal networks (Ibrahim et al. 2020), and in its dysfunction when blocked by mGluR5 antibodies. The novel antibody against the DPPX antigen as a subunit of Kv4.2 potassium channels was proven via immunoprecipitation and mass spectrometry in four patients (Boronat et al. 2013) associated with agitation and confusion as psychiatric symptomatology (Fig. 1). Several novel antibodies associated with encephalitis have been reported in the last 5 years, such as synapsin and neurexin 3 alpha antibodies. Both synapsin as a vesicle-colocated protein at the synapse (Piepgras et al. 2015) and neurexin3alpha as a cell-adhesion protein (Gresa-Arribas et al. 2016) are important for regulating synaptic function. Thus, it is not surprising that confusion (Piepgras et al. 2015; Gresa-Arribas et al. 2016) and disorientation were observed as prominent symptoms in patients diagnosed with synapsin and neurexin 3 alpha encephalitis (Fig. 1). We have been aware of glycine-receptor antibodies for years, i.e., in stiff person spectrum disorders such as progressive encephalomyelitis with stiffness and rigidity (PERM). However, these antibodies were observed just 2 years ago in relation to the clinical appearance of cognitive dysfunction (Swayne et al. 2018) (Fig. 1). Serum and CSF IgLON5 antibodies provide another example, as they can induce an encephalitis often associated with cognitive dysfunction (Simabukuro et al. 2015) (Fig. 1) including figural memory deficits (Hansen et al. 2020a) in addition to neurological deficits. Serum and CSF KLP11 antibodies were recently described in young male patients presenting with ocular symptoms and vertigo together with a seminoma (Mandel-Brehm et al. 2019). These serum and CSF KLP11 antibodies were additionally reported to be associated with psychosis in a further investigation (Maudes et al. 2020), although the mechanistic basis of these antibodies generating psychosis is unclear (Fig. 1).

## Discussion

### Synopsis: psychiatric symptoms in subtypes of autoimmune encephalitis

Our review shows that, as identified through various CSF autoantibodies, specific types of autoimmunity are associated with a psychiatric spectrum comprising disorientation, cognitive and memory dysfunction, behavioral abnormalities, psychosis, mood dysfunction, anxiety, obsessive–compulsive behavior, catatonia and sleeping dysfunction. Suicidality in autoimmune encephalitis patients has only been reported in conjunction with serum VGKC and mGluR5 autoantibodies. This evidence suggests that there are no specific autoantibodies in autoimmune encephalitis patients associated with specific symptoms apart from CSF NMDA autoantibodies in catatonia, or serum Ma/Ta autoantibodies in obsessive–compulsive behavior. The psychiatric syndrome and symptoms in autoimmune encephalitis patients are associated with various autoantibodies. These data show that it would be worthwhile to test for a panel of autoantibodies and not just specific autoantibodies in patients presenting with psychiatric symptoms. Furthermore, it is mainly those autoantibodies against membrane surface antigens in encephalitis patients that are associated with psychiatric features, as these autoantibodies interfere with neurotransmission, and psychiatric symptoms are often caused by impaired neurotransmission.

### Prospective challenges

Our main challenge is to describe the psychiatric phenotype of autoimmune encephalitis patients associated with autoantibodies. There have been no large-scale prospective or retrospective studies investigating various autoantibodies. Discovering a specific psychiatric phenotype in patients with specific autoantibody-positive autoimmune encephalitis would be of major interest to enable specific and effective treatment for these patients. Further prospective challenges concern views of underlying immune pathophysiology of autoimmune encephalitis associated with psychiatric symptoms. More cause–effect studies are needed to better understand the role of autoantibodies. Prior studies involving the transfer of human autoantibodies to rodents ranging from α-amino-3-hydroxy-5-methyl-4-isoxazolepropionic acid receptor (AMPAR), glutamic acid decarboxylase 65 (GAD65), glycine- or leucine-rich glioma-inactivated 1 (LGI1) or NMDAR autoantibodies have demonstrated that neural transmission on the cellular level with consecutive, altered signal cascades could induce psychiatry symptoms and cognitive anomalies on the behavioral level (Haselmann et al. 2018; Planaguma et al. 2015; Jurek et al. 2019; Geis et al. 2012; Petit Pedrol et al. 2018). These animal model studies confirm that autoantibodies play a pathogenic role in inducing psychiatric and neurocognitive symptoms in autoimmune encephalitis patients. However, further studies should be conducted to elucidate the exact role of autoantibody subgroups and their correlation to psychopathology assessed via different psychometric approaches. In this sense, novel research tools for classification of psychopathology as the hierarchical taxonomy of psychopathology comprising spectra and subfactors (Ruggero et al. 2019; Kotov et al. 2020) apart from the standard assessment according to Diagnostic and Statistical Manual of Mental Disorders Fifth Edition (DSM V) are promising. Apart from the relationship between psychopathology and autoantibodies, the relationship between immune cell subsets as potential biomarkers of autoimmune encephalitis (Hansen et al. 2020b, c) is interesting, as a recent study demonstrated a potential role of cerebrospinal fluid B- and T-cell activity and cognitive dysfunction in suspected limbic encephalitis (Helmstaedter et al. 2020). Thus, we are interested in assessing CSF sets and subsets of immune cell populations via fluorescence-activated cell sorting analysis in patients with autoimmune encephalitis and predominant psychiatric symptoms to identify patterns of immune cells, thereby providing new insight into the pathophysiologic mechanisms generating psychiatric symptoms in autoimmune encephalitis. Seeking such immune-cell subsets is particularly worthwhile, as a recent study revealed that in NMDAR encephalitis, the clinical syndrome psychosis’ generation is not associated with NMDAR autoantibodies (Engen et al. 2020), suggesting that the role that NMDAR autoantibodies play in immunopathology is being overrated.

## Conclusions

As psychiatric symptoms and syndromes have been less intensively investigated than neurological deficits and symptoms in autoimmune encephalitis patients, these symptoms may be underdiagnosed in patients with autoimmune encephalitis: There is thus a great need for more detailed descriptions of psychiatric syndromes and symptoms in patients with autoimmune encephalitis to enable the psychiatric features of this disease to be better characterized, thereby ensuring these patients receive more effective therapy following a psychiatric onset or predominantly psychiatric manifestation of autoimmune encephalitis.
